# Evaluation of the Collection Efficiency of a Wet-Type Electrostatic Precipitator for Aerosolized Influenza A Virus

**DOI:** 10.3390/microorganisms14081863

**Published:** 2026-08-21

**Authors:** Kazuya Nakamura, Takeshi Nagai, Hitoshi Ishiguro, Keiichi Kobayashi, Kazuhisa Nakagawa, Masahiro Okanojo, Akira Nukazuka

**Affiliations:** 1 DENSO CORPORATION, 1-1 Showa-cho, Kariya 448-8661, Aichi, Japan; 2 Kanagawa Institute of Industrial Science and Technology, LiSE 4c-1, 3-25-13 Tonomachi, Kawasaki-ku, Kawasaki 210-0821, Kanagawa, Japan

**Keywords:** electrostatic precipitator, aerosolized influenza A virus, collection efficiency, bioaerosol sampling, airborne virus detection

## Abstract

Airborne viruses are a key driver of infectious disease transmission, highlighting the importance of reliable detection in public health surveillance. As atmospheric viral concentrations are very low, a sampler with a high viral collection efficiency is essential. Although multiple approaches for evaluating collection efficiency have been applied using various samplers, no standardized sampler has yet been developed. We previously developed a wet-type electrostatic precipitator (WT-ESP) and successfully collected severe acute respiratory syndrome coronavirus 2 from the public environment. However, its efficiency for quantitative collection of airborne viruses remains unclear. This study aimed to clarify the collection efficiency of the WT-ESP. We evaluated collection efficiency via two different approaches: direct spray, where virus-containing aerosols were sprayed directly into the WT-ESP inlet, and indirect spray, where aerosols were dispersed into a closed space and then collected using the sampler. The direct spray tests achieved 20.1–50.2% collection efficiency, whereas the indirect spray test achieved an efficiency <12%. These findings highlight that electrostatic precipitation has an advantage of enhancing collection efficiency compared with values reported for impingers in previous studies and provide preliminary insights into the collection efficiencies under direct and indirect spray conditions, providing foundational data that bridge the gap between both methods.

## 1. Introduction

Viruses have long posed considerable threats to public health and the global economy. The major virus transmission routes include contact, droplets, and airborne transmission. These allow viruses to spread easily through daily actions, such as coughing, sneezing, and conversation, potentially leading to widespread outbreaks [1,2,3,4,5,6,7]. In recent years, large-scale infectious disease outbreaks, such as the 2003 severe acute respiratory syndrome (SARS) outbreak, 2009 H1N1 influenza pandemic, 2015 H5N2 avian influenza outbreak, and 2019 severe acute respiratory syndrome coronavirus 2 (SARS-CoV-2) pandemic, have severely impacted both human populations and the livestock industry [8,9,10,11]. Therefore, early detection of the virus is critical for preventing the spread of infection. However, enhancing the screening of at-risk individuals or livestock is impractical because of cost and labor constraints [12]. In contrast, airborne viral detection offers a scalable approach for early detection and is a promising solution for preventing pandemics. The concentrations of airborne viruses, such as influenza and SARS-CoV-2, have been reported in previous studies. For example, airborne influenza viruses and SARS-CoV-2 are reported to exist at levels of 10^1^–10^5^ copies/m^3^ [12,13,14,15] and 0.1–10^6^ copies/m^3^ [16,17] during hospital outbreaks, respectively. Moreover, airborne SARS-CoV-2 concentrations in cars were reported to be 10^3^–10^4^ copies/m^3^ [18]. Considering that the detection limit of widely used polymerase chain reaction (PCR)-based assays is approximately 1 copy/μL [19,20], a condensation ratio > 10^4^–10^9^ is required for detection, indicating that a high collection efficiency is essential.

Various bioaerosol samplers, such as impactors, impingers, cyclones, filters, and electrostatic precipitators (ESPs), have been reported as concentrators of airborne viruses in laboratory settings [21,22]. The collection mechanisms of these samplers are based on various physical principles. Impactors and impingers utilize inertial forces to separate particles from the airstream and deposit them on solid and liquid surfaces, respectively. Cyclones use centrifugal forces to drive particles toward the chamber wall. Filters capture particles primarily via interception, inertial impaction, diffusion, and electrostatic attraction. ESPs use electrostatic forces to attract and collect charged particles onto electrodes [23,24]. In addition to conventional filtration technologies, ESPs have recently attracted considerable attention as an energy-efficient approach for removing airborne particulate matter and bioaerosols. Recent studies have demonstrated that electrostatic precipitators can achieve effective particle removal while maintaining a low-pressure drop, making them promising alternatives to conventional fibrous filtration systems [25]. In ESPs, particles are typically charged by a corona discharge and subsequently transported toward collection electrodes by an electric field. This mechanism enables efficient collection of submicron particles with a minimal pressure drop, making ESPs suitable for continuous aerosol sampling [26]. The collection efficiencies of these samplers have been compared in laboratory settings [27,28].

The method for spraying virus-containing aerosols can be categorized into two types: direct spraying of aerosolized viruses into a sampler’s inlet (direct spray method) [27] and indirect spraying where aerosols are dispersed into the air before collecting airborne viruses with bioaerosol samplers (indirect spray method) [28]. To assess the intrinsic collection efficiency of a sampler, the direct spray method is appropriate because it does not depend on the size of the spraying space. In contrast, the indirect spray approach better reproduces real-world operating conditions, as it involves aerosol transport and dispersion during actual use. As aerosolized viruses behave differently between these two spray methods, the collection efficiency also differs between them [27,29]. However, quantitative comparisons of these two methods are limited.

In this study, we used a wet-type ESP (WT-ESP) originally developed by Fukuda et al. [30]. The WT-ESP is a promising sampler for submicron-sized viral collection because ESPs offer a high collection efficiency for fine particles [30,31,32,33]. A previous study succeeded in collecting SARS-CoV-2 in hospitals and public spaces, such as food courts and offices. However, quantitative evaluation of the collection efficiency of airborne viruses has not yet been performed. Therefore, this study aimed to assess the collection efficiency of the WT-ESP for aerosolized viruses. In particular, we addressed the gap in collection efficiencies between the direct and indirect spray methods by conducting a controlled comparison. In the direct spray method, influenza A virus (H3N2) was sprayed directly into the WT-ESP inlet, whereas in the indirect spray method, the same viral species was sprayed within a closed 375-L chamber and collected using the WT-ESP. For precise comparison, we considered the natural decrease in aerosolized viruses caused by deposition and sedimentation in the indirect spray method.

## 2. Materials and Methods

### 2.1. Reagents and Materials

The following reagents and materials were used: Eagle’s minimum essential medium (EMEM), 100× mixture of penicillin/streptomycin, phosphate-buffered saline (PBS), formaldehyde, and trypsin were obtained from FUJIFILM Wako Pure Chemical Corporation (Osaka, Japan). Fetal bovine serum (FBS), 100× non-essential amino acid (NEAA), and sodium bicarbonate (NaHCO_3_) were purchased from Sigma-Aldrich (St. Louis, MO, USA). Agar was obtained from Ina Food Industry (Nagano, Japan) and DEAE-dextran from pK Chemicals (Horsholm, Denmark). Methylene Blue was purchased from WALDECK (Münster, Germany) for cell staining. Virus collection buffer consisted of PBS supplemented with 3% bovine serum albumin, 0.1 mg/mL transfer ribonucleic acid (tRNA), 3 mM magnesium chloride, and 0.1% (*v*/*v*) Tween-20, all of which were purchased from FUJIFILM Wako Pure Chemical Corporation. The QIAamp Viral RNA Mini Kit and QuantiTect Probe RT-qPCR Kit were purchased from Qiagen (Hilden, Germany). The primer/probe set for influenza A virus detection (catalog no. RC500A) was obtained from Takara Bio (Shiga, Japan).

### 2.2. Virus and Host Cells

In this study, we used H3N2 as a model airborne respiratory virus because it can be handled safely under our experimental conditions and its infectivity can be quantitatively evaluated using established cell-based assays, such as the plaque assay. H3N2 (VR-1679) and its host Madin–Darby Canine Kidney (MDCK, CCL-34) cells were purchased from the American Type Culture Collection (Manassas, VA, USA). MDCK cells were maintained in growth medium (EMEM supplemented with 10% heat-inactivated FBS and 1× mixture of NEAA and penicillin/streptomycin) at 37 °C in a 5% CO_2_ atmosphere. All experiments were performed in a biosafety level 2 laboratory.

### 2.3. Preparation of Viral Suspension

MDCK cells were seeded in a T-75 flask containing 20 mL of growth medium and cultured to 95% confluence. After removing the medium and washing the cells with 10 mL of PBS, 1 mL of the viral suspension was inoculated onto a monolayer of host cells at a multiplicity of infection of 0.001. After incubating the cells for 1 h at 34 °C, 10 mL of FBS-free EMEM supplemented with 25 μg/mL of trypsin was added. The cells were cultured for 4 days at 34 °C in the presence of 5% CO_2_. When a sufficient cytopathic effect was observed, 10 mL of the culture medium was collected into a 15 mL tube and centrifuged at 1000× *g* for 20 min at 4 °C. The supernatant was collected into cryotubes and stored at −80 °C as a viral suspension for aerosolization. The infectious titer of the virus was approximately 1.0 × 10^8^ plaque-forming units (PFU)/mL.

### 2.4. Bioaerosol Sampler Design

**Figure 1:**
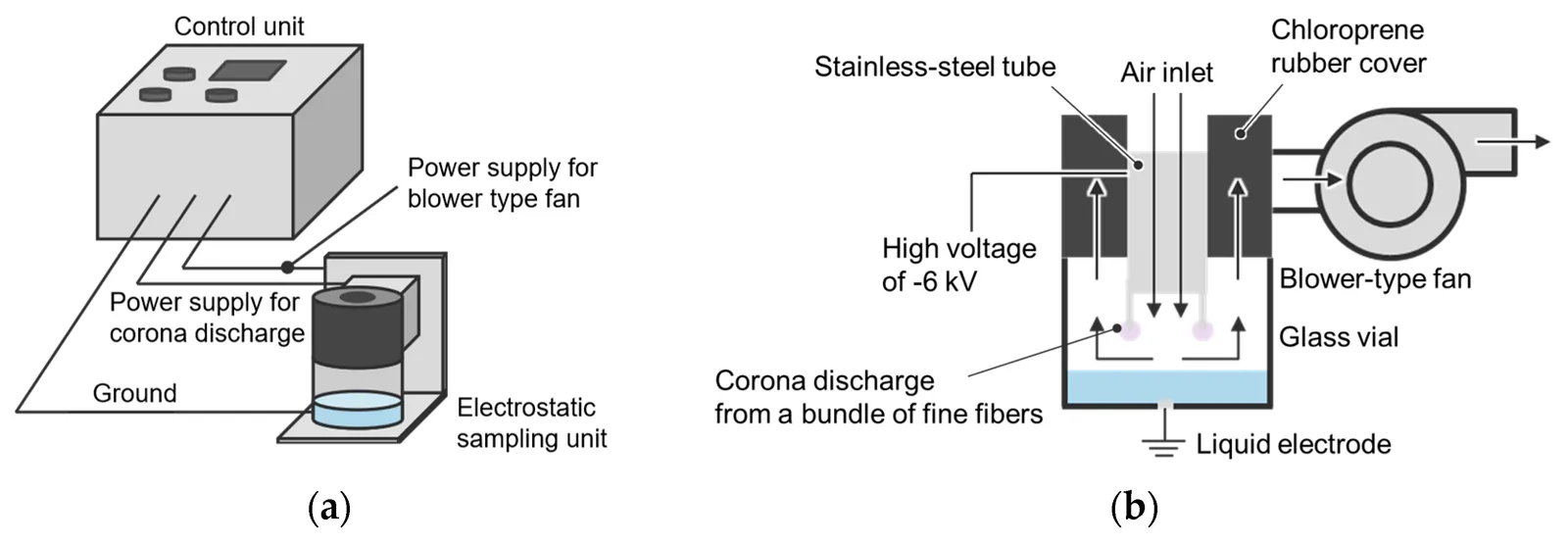
Design of the sampler. (**a**) An overview of the aerosol sampler composed of two units: the electrostatic sampling and control units. (**b**) A schematic diagram of the electrostatic sampling unit. Black arrows indicate the flow of air.

The WT-ESP method reported by Fukuda et al. [30] was used as a reference and modified for aerosol sampling in this study. The electrostatic sampling unit (Figure 1) consisted of a blower-type fan, a commercially available glass vial (inner diameter: φ40 mm), and a chloroprene rubber cover with a stainless-steel tube at the center (inner diameter: φ20 mm) and 10 holes arranged around the tube. The multiple stainless-steel fiber (100 fibers with fiber diameters of 12 µm) electrodes were attached to the tube. The glass vial contained 4 mL of virus collection buffer, which was used as a liquid electrode. To collect the aerosolized viruses, the applied voltage, airflow rate, and electrode–liquid distance of the sampler were set to −6 kV (negative corona discharge), 40 L/min, and 7 mm, respectively, consistent with the previously reported conditions [30]. The discharge current ranged from 150 to 200 µA throughout the experiments, corresponding to an electrical power consumption of approximately 0.9–1.2 W. This variation was attributed to evaporation of the liquid electrode at a rate of approximately 50 µL/min. The 5 V/0.4 A blower fan was positioned downstream of the collection chamber to establish airflow through the electrostatic sampling unit. Air entered the sampler through the central stainless tube, passed through the corona discharge region, and subsequently flowed across the liquid collection surface before being exhausted through the 10 holes in the chloroprene rubber cover and blower-type fan outlet. Ozone concentration and electric field strength were not measured in this study.

### 2.5. Experimental Setup and Workflow

**Figure 2:**
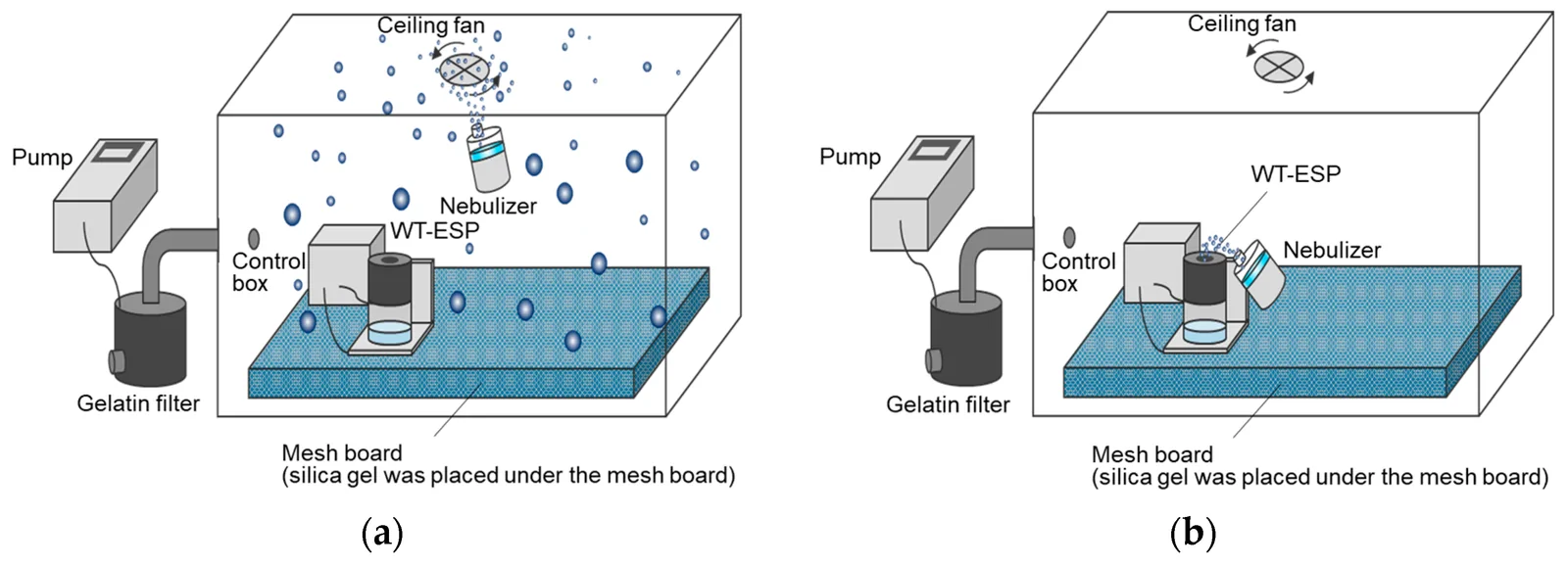
Two types of spray methods. (**a**) In the indirect spray tests, the nebulizer sprayed the viral suspension under the ceiling fan and dispersed the aerosols into the air. (**b**) In the direct spray test, the nebulizer sprayed the suspension directly to the inlet of the wet-type electrostatic precipitator.

Aerosolization of the influenza A virus was conducted inside a flow-type glove box (Sanplatec, Oosaka, Japan, G-1000S, 375 L), as shown in Figure 2. Prior to the experiment, silica gel was placed at the bottom of the glove box to regulate humidity. The internal humidity was maintained at 9–12% throughout the experiment. A mesh board was installed above the silica gel, and the aerosol sampler was placed on it. Additionally, a ceiling-mounted fan continuously stirred the air inside the chamber to ensure a uniform virus distribution. A mesh-type nebulizer (Omron, Kyoto, Japan, NE-U22) was used for aerosol generation. A gelatin filter, which was placed outside the glove box, was connected to the glove box through polyvinyl chloride tubing, and air was drawn using a portable sampler (MD8 Airport; Sartorius AG, Göttingen, Germany).

We conducted two types of experiments: the direct and indirect spray tests. Figure 2a shows the indirect spray test. In this test, 1.0 mL of viral suspension was loaded into the nebulizer, sprayed closely beneath the fan, and dispersed into the air. During this process, the WT-ESP remained off until aerosolization ceased. After aerosolization, the WT-ESP was activated, and the aerosolized viruses in the air were collected for 10 min. Figure 2b shows the direct spray test. Here, 1.0 mL of viral suspension in the nebulizer was sprayed near the WT-ESP until no aerosols emerged from the nebulizer. For both test types, a small volume of the suspension remained in the nebulizer. Therefore, we subtracted the remaining volume of the suspension from the initial 1.0 mL of viral suspension to calculate the total volume of the sprayed aerosol.

The collected virus solution was transferred into a 15 mL tube placed in an aluminum container inside the glove box. UV radiation was then applied for 15 min to inactivate viruses within the box. The UV shielding effect of the aluminum container was confirmed using a UV radiometer. Finally, the aluminum container was moved from the glove box to a safety cabinet, and the infectious titer and copy number of the recovered viruses were analyzed using the plaque assay and RT-qPCR, respectively.

### 2.6. Plaque Assay

The titer of the influenza A virus was determined using a plaque assay technique, as referenced by ISO 21702 [34,35]. Briefly, 0.1 mL of the collected viral suspension, as well as a suspension serially diluted 10-fold in EMEM, was inoculated onto monolayered MDCK cells in 6-well culture plates. The plates were incubated for 1 h at 34 °C with gentle rocking every 15 min. Thereafter, EMEM containing 0.75% agar, 0.15% NaHCO_3_, 0.01% DEAE-dextran, and 25 μg/mL of trypsin was added. After solidification, the plates were incubated for 4 days at 34 °C in the presence of 5% CO_2_ and then 3.7% formaldehyde aqueous solution was added to every well for fixation. After removing the agar medium, the MDCK cells were stained with Methylene Blue, and the number of plaques was counted. The plaque assay was duplicated in each test, and the PFU values were calculated using the number of plaques and dilution ratio.

### 2.7. RT-qPCR

RT-qPCR of the influenza A viral genome was performed according to the protocols of the Japanese National Institute of Infectious Diseases [36]. Briefly, 140 μL of the collected viral suspension was used to extract viral RNA using the QIAamp Viral RNA Mini Kit, which was then eluted in 60 μL of the elution buffer. Next, 5 μL of the eluted RNA sample was evaluated for viral load using the QuantiTect^®^ Probe RT-qPCR Kit on a LightCycler^®^ 480 System II (Roche Diagnostics, Basel, Switzerland). The nucleoprotein gene of the influenza A virus was amplified using a commercial primer/probe set for influenza A detection. Reverse transcription was performed at 55 °C for 10 min, followed by initial denaturation at 95 °C for 3 min. PCR amplification was then carried out for 45 cycles at 95 °C for 15 s and 58 °C for 30 s. Quantification was performed according to the manufacturer’s instructions.

### 2.8. Evaluation of Spatial Residual Ratio of Aerosolized Viruses

To accurately compare the collection efficiency of viruses using direct and indirect sampling, we evaluated the spatial residual ratio of the aerosolized viruses. After viruses were dispersed inside the glove box, 10 L of air in the glove box was collected by gelatin filter at three time points: immediately after the dispersion was completed (0 min) and 30 and 60 min after the completion of the dispersion. A plaque assay was conducted at each time point. The spatial residual ratio is defined by the following equation:

(1) $$SRR\left(t\right)=\frac{C_{plaque}\left(t\right)}{C_{plaque}\left(0\right)}$$

where SRR refers to the spatial residual ratio, t is the time when the viral dispersion was completed, and $C_{plaque}$ denotes the virus concentration quantified by the plaque assay.

### 2.9. Evaluation of Collection Efficiency

The collection efficiency of viruses by the WT-ESP was evaluated using the following equations

(2) $$\eta_{direct}=\frac{C_{collected}\times V_{rec}}{C_{initial}\times V_{aero}}\times 100$$

(3) $$\eta_{indirect}=\frac{C_{collected}\times V_{rec}}{C_{initial}\times V_{aero}\times SRR(t)}\times 100$$

$\eta_{direct}$ and $\eta_{indirect}$ refer to the viral collection efficiency by the direct and indirect spray methods, respectively. $C_{collected}$ is the concentration of the collected viruses in the sampling buffer, which was quantified by plaque assays or RT-qPCR. $C_{initial}$ indicates the initial virus concentration before aerosolization. $V_{rec}$ and $V_{aero}$ are the volumes of the recovered collection liquid and the aerosolized virus suspension, respectively. It should be noted that the correction in Equation (3) assumes that the airborne concentration during indirect sampling follows the sampler-off decay curve. Since the running sampler itself removes virus-laden air in the indirect spray tests, the applied SRR could be overestimated, and $\eta_{indirect}$ calculated using Equation (3) could consequently be underestimated. We did not conduct a statistical analysis in this study because the experimental workload per run was high and we could not obtain enough data points for statistical analysis. Therefore, data have been interpreted qualitatively.

## 3. Results

### 3.1. Virus Collection Efficiency of the WT-ESP in the Direct Spray Method

The virus collection efficiency of the sampler using the direct spray method was evaluated. Initial titers and copy numbers of the sprayed virus ranged from 1.7 × 10^4^ to 2.2 × 10^8^ PFU/mL and 5 × 10^3^ to 7.6 × 10^7^ copies/μL, respectively.

**Figure 3:**
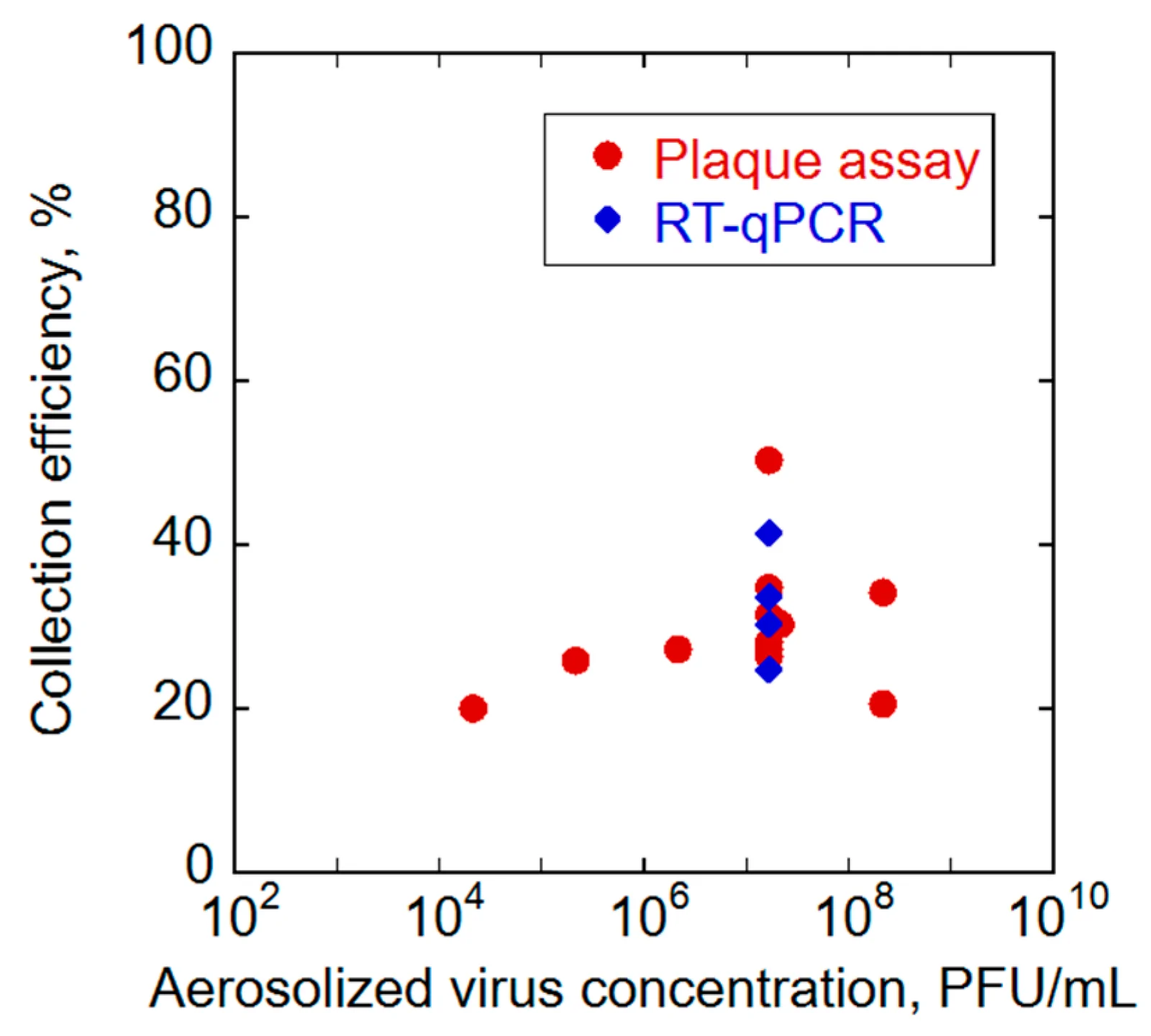
Results of the influenza A virus collection efficiency by the direct spray method.

The collected viral suspension was quantified by plaque assays and RT-qPCR, and the collection efficiencies were calculated using Equation (2). The collection efficiencies ranged from 20.1% to 50.2% across the tested virus concentrations (Figure 3). At an aerosolized virus concentration of 1.7 × 10^7^ PFU/mL and 2.2 × 10^8^ PFU/mL, the collection efficiency ranged from 26.4% to 50.2% by RT-qPCR and 20.6% to 34.1% by plaque assay, respectively. At lower concentrations of 2.2 × 10^4^, 2.2 × 10^5^, and 2.2 × 10^6^ PFU/mL, the collection efficiencies were 20.1%, 25.8%, and 27.3%, respectively. Within the limited number of replicates in this study, no clear concentration-dependent trend in collection efficiency was observed. The virus collection efficiency evaluated by RT-qPCR was assessed at an aerosolized virus concentration of 1.7 × 10^7^ PFU/mL (corresponding to 4.3 × 10^6^ copies/μL) and it ranged from 26.4% to 41.5%. This range was within the collection efficiency obtained by the plaque assay, indicating no substantial difference in collection efficiency between RT-qPCR and the plaque assay.

### 3.2. Virus Collection Efficiency of the WT-ESP in the Indirect Spray Method

#### 3.2.1. Evaluation Results of Spatial Residual Ratio

**Figure 4:**
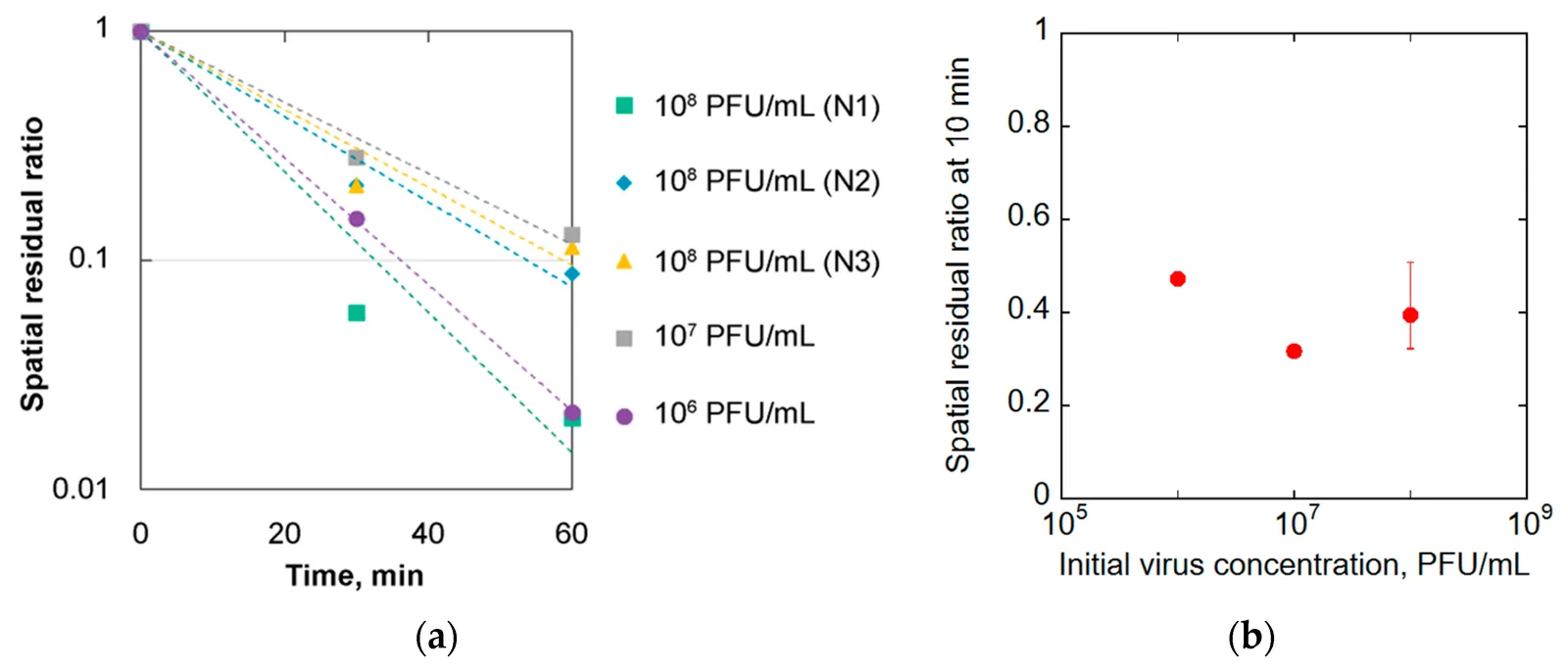
(**a**) Experimental results of spatial residual ratio at each time point. Dotted lines indicate the exponential regression curves fitted for each SRR data. The 10 min SRR values used for collection-efficiency calculations were estimated from these fitted curves. Data for the sprayed concentrations of 10^6^, 10^7^, and 10^8^ PFU/mL were obtained with *n* = 1, 1, and 3 independent experimental replicates, respectively. (**b**) The spatial residual ratio at 10 min for each sprayed concentration was estimated by interpolation from the exponential fitting curves. Error bars represent the standard deviation of the measurements for 10^8^ PFU/mL (*n* = 3).

To compare the collection efficiencies of the direct and indirect spray methods, we evaluated the spatial residual ratio of aerosolized viruses in the chamber. Airborne virus decay occurs via processes, such as gravitational settling, deposition onto the surfaces of the glove box, and virus inactivation caused by drying, all of which reduce airborne virus levels independent of sampler performance. The sprayed concentrations were 10^6^, 10^7^, and 10^8^ PFU/mL, with replicate numbers of 1, 1, and 3, respectively. All samples were quantified using plaque assays. The spatial residual ratio calculated using Equation (1) is shown in Figure 4a. At an initial viral concentration of 10^8^ PFU/mL, the spatial residual ratio dropped approximately to 0.15–0.30 at 30 min and to 0.08–0.12 at 60 min. At lower initial concentrations of 10^6^ and 10^7^ PFU/mL, a similar time-dependent decay trend was observed. At initial concentrations of 10^6^ and 10^7^ PFU/mL, the spatial residual ratio was 0.15 and 0.28 at 30 min, and 0.02 and 0.13 at 60 min, respectively. All the data exhibited a linear trend on a logarithmic scale for all viral concentrations. Although extending the sampling duration is expected to increase recovery efficiency, it also leads to greater natural decay of airborne viruses and increases variability among experiments. These data suggest that the spatial residual ratio is essential for comparing the indirect and direct spray methods.

In the indirect spray method, the sampling time was set to 10 min for two reasons. First, a 10 min sampling period minimizes natural decay and variability. Second, sampling at a flow rate of 40 L/min for 10 min corresponds to a nearly complete inhalation of the air volume within the 375-L glove box. Given that the virus concentration was uniformly distributed, 10 min sampling could be considered as the time to inhale the whole air in the glove box. The 10 min spatial residual ratio used for collection efficiency calculations was estimated from exponential regression curves fitted to the SRR data obtained at 0, 30, and 60 min. Regression analysis was performed separately for each virus-concentration condition, and the resulting equations were used to calculate the corresponding 10 min SRR values. The estimation results are shown in Figure 4b. The spatial residual ratio at 10^8^ PFU/mL ranged from 0.32 to 0.50. The rates at 10^6^ and 10^7^ PFU/mL were 0.47 and 0.32, respectively, both of which were within the variability range of 10^8^ PFU/mL. The mean spatial residual ratio across all experiments was 0.39 at 10 min. In the subsequent indirect spray test, this value was used to calculate the collection efficiency of the WT-ESP sampler. It should be noted that viral decay behavior may depend on viral concentration. Therefore, using a single average spatial residual ratio represents a limitation of this study. Further concentration-dependent SRR analysis should be addressed in future studies.

#### 3.2.2. Evaluation of Virus Collection Efficiency of the WT-ESP by the Indirect Spray Method

After determining the spatial residual ratio, the virus collection efficiency of the sampler was evaluated using the indirect spray method. The initial titers and copy numbers of the sprayed viruses were the same as those used for the direct spray method. The collected virus solution was quantified by plaque assays and RT-qPCR, and the collection efficiencies were calculated using Equation (3), which considers the spatial residual ratio to be 0.39.

**Figure 5:**
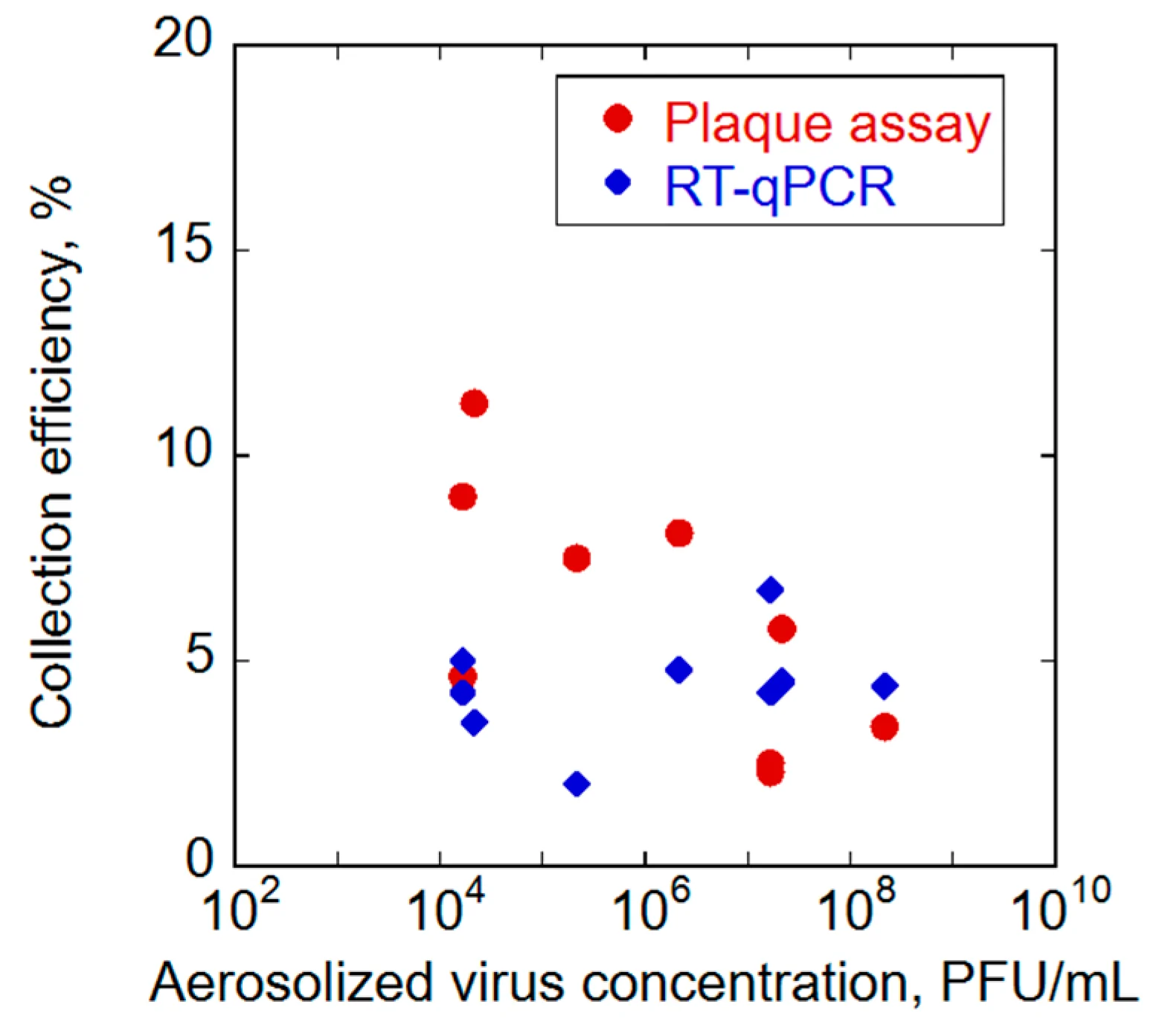
Results of the influenza A virus collection efficiency by the WT-ESP in the indirect spray method.

At an initial aerosolized virus concentration of 1.7 × 10^4^ PFU/mL, the collection efficiencies ranged from 4.6 to 11.3% by plaque assay (Figure 5). At higher aerosolized viral concentrations of 2.2 × 10^5^, 2.2 × 10^6^, 2.2 × 10^7^, and 2.2 × 10^8^ PFU/mL, the collection efficiencies were 7.5%, 8.1%, 5.8%, and 3.4%, respectively. At 1.7 × 10^4^ PFU/mL, the collection efficiencies ranged from 3.5 to 5.0% by RT-qPCR. At 2.2 × 10^5^, 2.2 × 10^6^, 2.2 × 10^7^, and 2.2 × 10^8^ PFU/mL, the corresponding values were 2.0%, 4.8%, 4.5%, and 4.4%, respectively. Within the limited replicate dataset, collection efficiencies did not show an obvious concentration-dependent trend. The indirect spray method exhibited a lower collection efficiency than the direct spray method.

In conclusion, the collection efficiency of the indirect spray method was approximately 4–6 times lower than that of the direct spray method, regardless of whether the measurement method was a plaque assay or RT-qPCR. Because we considered the spatial residual ratio in the calculation of collection efficiency (Equation (3)), the large drop in collection efficiency in the indirect spray test was attributed to the difficulty of collecting viruses distributed over the glove box.

## 4. Discussion

In this study, the WT-ESP exhibited different characteristics from those reported in previous studies using conventional impingers, particularly with respect to collection efficiency. Previous studies using impingers reported that the collection efficiencies of influenza A virus under direct spray conditions ranged from of 0.5–5% [27]. In contrast, the average collection efficiencies of the WT-ESP under the same direct and indirect spray conditions were 32.8% and 5.2%, respectively, indicating that a higher collection efficiency was achieved herein. This improvement is likely attributable to the electrostatic capture mechanism, which enhances the collection of charged aerosolized viruses without relying on the inertial impact on a liquid surface, as in conventional impingers. To assess the impact of uncertainty on the collection efficiency of the indirect spray associated with viral decay, we additionally considered the variability in the 10 min residual ratio. As shown in Figure 4a, the resulting collection efficiency was estimated to range from 4.7% to 7.6%. Importantly, even the lower limit of this range remains higher than the collection efficiencies reported for conventional impingers in previous studies [33]. In addition, the potential effects of environmental humidity, liquid buffer formulation, and fan-induced mixing on collection efficiency were not systematically evaluated in this study and should be investigated in future work. In particular, the relative humidity in this study (9–12%) was much lower than that typically maintained in indoor environments such as hospitals or offices (40–60%). Such low humidity could substantially affect virus survival and aerosol particle size during indirect spray experiments, highlighting the importance of evaluating the influence of humidity when interpreting collection efficiency under real-world indoor conditions.

In addition to the enhanced collection efficiency, the WT-ESP exhibited another characteristic distinct from impingers in terms of viral infectivity. Generally, ESPs have been reported to reduce viral infectivity because the radicals generated by corona discharge can damage viral nucleic acids and proteins [37,38]. Considering that previous studies reported virus inactivation under the same −6 kV corona discharge conditions [38,39], one possible explanation is the relatively short sampling duration used in this study. However, the contribution of corona discharge-induced viral inactivation was not directly evaluated and therefore remains uncertain, highlighting the need for further studies to clarify the extent of viral inactivation under these conditions.

Together, these results show that the direct spray method demonstrates high collection efficiency and reproducibility, making it a favorable method for evaluating viral behavior. Although the indirect spray method is useful for simulating indoor environmental exposure, its collection efficiency was substantially lower than that of the direct spray method. Overall, the spatial residual ratio obtained in this study, along with the discussion of the characteristics of each method, provides insights that contribute to optimizing virus collection methods and refining evaluations in real-world environments. Even if the collection efficiency of the WT-ESP is higher than conventional impingers, it still needs to be improved. For real-world monitoring applications, sampling performance may be improved by increasing the air flow rate and/or the number of samplers deployed within a monitored space. Strategic placement of samplers in areas where airborne particles are likely to accumulate because of limited air circulation may further enhance detection probability. Furthermore, the use of low-volatility collection media, such as ionic liquids, may help maintain stable collection performance during long-term operation. However, further analyses, such as those determining the influence of chamber size, the contribution of each viral loss pathway in the indirect spray test, and the applicability of this method to different types of viruses, such as SARS-CoV-2, are needed because these factors may affect airborne viral behavior. For example, if the chamber volume increases ten-fold, the surface area increases according to the two-thirds power of the volume, potentially resulting in nonlinear effects on collection efficiency. Additionally, although no obvious concentration-dependent trend was observed in the tested concentration range, owing to the limited number of replicates, this should be regarded as a qualitative observation rather than evidence of concentration independence.

In conclusion, this study clarified the quantitative difference between direct and indirect aerosol virus sampling using the WT-ESP. Using the direct spray method, we achieved high collection efficiency of airborne viruses. Conversely, in the indirect spray method, the collection efficiencies reduced markedly to 15–25% of the values by the direct spray method. These reductions reflect the difficulty of drawing airborne viruses distributed in large spaces into the sampler. As the virus collection efficiency was evaluated only in a 375-L space in this study, further investigation of the dependence on space volume is required. The quantitative relationship of collection efficiency between the direct and indirect spray tests established here provides a practical reference for interpreting sampling data under different aerosolization conditions and supports the use of ESP for accurate environmental virus monitoring.

## Data Availability

The original contributions presented in this study are included in the article. Further inquiries can be directed to the corresponding authors.

## Abbreviations

WT-ESP	wet-type electrostatic precipitator
SARS	severe acute respiratory syndrome
SARS-CoV-2	severe acute respiratory syndrome coronavirus 2
EMEM	Eagle’s minimum essential medium
PBS	phosphate-buffered saline
NEAA	non-essential amino acid
MDCK	Madin–Darby Canine Kidney
PFU	plaque-forming units
